# Stroke-GFCN: ischemic stroke lesion prediction with a fully convolutional graph network

**DOI:** 10.1117/1.JMI.10.4.044502

**Published:** 2023-07-17

**Authors:** Ariel Iporre-Rivas, Dorothee Saur, Karl Rohr, Gerik Scheuermann, Christina Gillmann

**Affiliations:** a Leipzig University, Institute for Computer Science, Faculty of Mathematics and Computer Science, Signal and Image Processing Group, Leipzig, Germany; b Max-Plank-Institute for Human Cognitive and Brain Sciences, Leipzig, Germany; c ScaDS.AI, Leipzig, Germany; d Leipzig University, Department of Neurology, Leipzig, Germany; e Heidelberg University, BioQuant Center, IPMB and DKFZ, Biomedical Computer Vision Group, Heidelberg, Germany

**Keywords:** medical imaging, stroke prediction, machine learning, graph neural networks, multi-modal imaging

## Abstract

**Purpose:**

The interpretation of image data plays a critical role during acute brain stroke diagnosis, and promptly defining the requirement of a surgical intervention will drastically impact the patient’s outcome. However, determining stroke lesions purely from images can be a daunting task. Many studies proposed automatic segmentation methods for brain stroke lesions from medical images in different modalities, though heretofore results do not satisfy the requirements to be clinically reliable. We investigate the segmentation of brain stroke lesions using a geometric deep learning model that takes advantage of the intrinsic interconnected diffusion features in a set of multi-modal inputs consisting of computer tomography (CT) perfusion parameters.

**Approach:**

We propose a geometric deep learning model for the segmentation of ischemic stroke brain lesions that employs spline convolutions and unpooling/pooling operators on graphs to excerpt graph-structured features in a fully convolutional network architecture. In addition, we seek to understand the underlying principles governing the different components of our model. Accordingly, we structure the experiments in two parts: an evaluation of different architecture hyperparameters and a comparison with state-of-the-art methods.

**Results:**

The ablation study shows that deeper layers obtain a higher Dice coefficient score (DCS) of up to 0.3654. Comparing different pooling and unpooling methods shows that the best performing unpooling method is the proportional approach, yet it often smooths the segmentation border. Unpooling achieves segmentation results more adapted to the lesion boundary corroborated with systematic lower values of Hausdorff distance. The model performs at the level of state-of-the-art models without optimized training methods, such as augmentation or patches, with a DCS of 0.4553±0.0031.

**Conclusions:**

We proposed and evaluated an end-to-end trainable fully convolutional graph network architecture using spline convolutional layers for the ischemic stroke lesion prediction. We propose a model that employs graph-based operations to predict acute stroke brain lesions from CT perfusion parameters. Our results prove the feasibility of using geometric deep learning to solve segmentation problems, and our model shows a better performance than other models evaluated. The proposed model achieves improved metric values for the DCS metric, ranging from 8.61% to 69.05%, compared with other models trained under the same conditions. Next, we compare different pooling and unpooling operations in relation to their segmentation results, and we show that the model can produce segmentation outputs that adapt to irregular segmentation boundaries when using simple heuristic unpooling operations.

## 1 Introduction

In the emergency room, physicians use neuroimaging to assess changes in blood irrigation in the brain and to define treatments.^1^ In particular, perfusion imaging is used to quantify the core and penumbra of the lesion in ischemic stroke patients and hereby tailor the treatment decision based on standardized procedures, as well as to predict effectiveness. Studies show that diffusion and perfusion weighted imaging based on magnetic resonance (DWI-MRI and PWI-MRI) are highly accurate methods for inferring the infarct core of the ischemic lesion.^2^^–^^4^ These techniques are very sensitive to intracellular water shifts after cell depolarization; hence it is easy to identify the core lesion.^5^ In practice, these scanners are often unavailable, or their acquisition time exceeds the time frame to extend a diagnosis to the patient,^6^ and computer tomography (CT) methods using contrast agents are preferred. Diagnosis using computer tomography perfusion (CTP) is rated as of equivalent value in trial and medical practice;^7^ nonetheless, CTP imaging is inherently more difficult to interpret because CTP is less sensitive to the small change in attenuation caused by water uptake in the acute ischemic brain tissue. In addition, apparent diffusion coefficient contrast increases linearly over time, and the image contrast changes at different time points during the diagnosis time. We are interested in improving the image analysis by making use of a graph neural network to automatically segment stroke lesions from CTP-parameter maps.

Imaging is essential in modern medical practice among all different specializations, but its analysis has many difficulties. Practitioners use them to assess the dimensions, structures, and topology of internal organs to identify abnormalities and establish a medical treatment. Although trained professionals can evaluate the patient’s condition from unprocessed images, the precise delimitation of relevant components remains difficult and time consuming. Studies confirm that there exists an intra- and inter-observer variability of manual segmentation that depends on the complexity of the target structure.^8^^–^^10^ These discrepancies could be associated with the intrinsic limitations of the cognitive processes in the human vision system.^11^ Moreover, in the case of a stroke diagnosis, many highly debated factors make harder the estimation of final infarct lesions for perfusion imaging methods. For example, reversal of the core and penumbra is sometimes observed, albeit the reported results were regarded as non-clinically significant.^12^^,^^13^ The extension of collateral vascularization, genetics, and external stimuli (e.g., chronic hypoperfusion) leads to changes in preliminary estimations of volumes mismatch estimations.^6^ Another factor, the lack of standardized software for the calculation of stroke parameters encumbers the definition of an optimized threshold value to make a simple threshold segmentation.^14^^–^^16^ In addition, the location of the ischemic stroke lesion affects the vulnerability of hypoperfusion and the outcome of the treatment.^15^ All of these difficulties make the estimation of penumbra from CTP a burdensome task, and a successful manual segmentation of stroke lesion from CTP images depends greatly on the expertise and ability of the interpreter. As a result, in an ischemic stroke diagnosis, manual segmentation is unpractical and prone to errors. In this regard, automated segmentation methods demonstrated promising potential to support the diagnosis of stroke patients.

In recent years, machine learning methods have been applied to solve the stroke lesion segmentation from CTP images problem with improved, yet not perfect, results.^17^^–^^19^ In general, the segmentation of brain structures from MRI or CT images is a difficult task and entails a considerable number of problems. For example, training machine learning models requires large amounts of data,^20^ but available datasets have only a few samples.^10^^,^^21^^–^^23^ Likewise, medical conditions, such as tumors, oedemas, and other lesions, introduce other problems such as fuzzy boundaries and have a high variance of shapes and locations.^19^^,^^24^^,^^25^ Additionally, imaging artifacts, different scanners/protocols, and anatomical variability (e.g., age and neurodegeneration) introduce contrast and intensity variations that also affect the stroke lesion datasets.^26^ Despite the success of convolutional neural networks (CNNs) in medical image analysis, in the case of ischemic stroke, there are many issues not thoroughly solved,^17^ and it is an active research area. Currently, the best solution is proposed in Ref. 27, which combines a generative model that produces a pseudo-DWI from the CTP-parameters and an attention-based loss. Additionally, other relevant solutions use U-Net on patches or 3D-convolutions in Refs. 28 and 29, respectively. However, the domain of CNN is a Euclidean domain, i.e., pixel grids, meaning that the structure of the features is limited by pixel position.^30^^,^^31^

An emerging field in deep learning denominated “geometric deep learning” proposes an extension of CNN to non-Euclidean structured convolution, which allows for positioning of inter-pixel features. Geometric deep learning has proven to successfully solve image classification of natural 2D images using spectral and spatial non-Euclidean convolutions.^32^^,^^33^ In neuroscience, graph neural networks have also been extensively used for the analysis of cortical gyrification: modeling of anatomical features,^34^ cortical parcellation,^35^^–^^38^ and understanding subtle topological dependencies in classification of functional MRI signals.^39^^–^^41^ Geometric deep learning leverages non-Euclidean convolution on meshes preserving cortical brain topology.^35^ In image segmentation, geometric deep learning is used to address the loss of feature localization.^42^^–^^44^ In a similar line to our proposed model, Juarez et al.^43^ and Lu et al.^44^ proposed graph fully convolutional network (GFCN) and U-Net-graph, respectively, to leverage the node connectivity but without using pooling operations. The absence of pooling introduces the disadvantage of increasing computational cost and memory footprint to process inputs. In addition, the mentioned models are based on spectral convolution, which lacks directional information and critical information to define the object boundaries.^32^ Our method differs from other graph encoder–decoder models,^44^^,^^45^ as we use spline convolution and pooling operators, allowing us to have different weights in different directions, thus extracting richer geometrical information.

In this work, we defined a deep learning model with graph convolutional operations in an encoder–decoder architecture for the task of segmentation of stroke lesions. To this end, we propose an architecture for graph-structured data that resembles the fully convolutional network (FCN),^46^ in which the convolution blocks are replaced with the spline convolutions proposed in Ref. 32, and upsampling layers are an approximation of interpolation in graphs. We theorize that a graph neural network could leverage from a more complex feature map and its capacity of connecting inter-pixel information in different angles to detect the lesion more accurately. We evaluated this by inferring the internal functionality of a graph-based CNN on the segmentation masks generated by our algorithm. The inputs to our model are non-contrast CT and CTP-parameters from the ischemic stroke lesion segmentation 2018 (ISLES2018) challenge dataset.^47^^,^^48^ We specifically study the flaws and benefits of using a geometric deep learning algorithm to predict ischemic tissue from CT-perfusion parameters and non-contrast CT in the ISLES2018 dataset.^47^^,^^48^ The ground truth corresponds to the core lesion; thus the model predicts irreversible lesion tissue probability. The model is trained under different configurations to extrapolate the internal processes of the model in correspondence to its hyperparameters. We compare the model against the reported results of Refs. 27–29. In addition, we train and compare the results of a U-Net,^49^ FCN-8s,^46^ and PointNet++.^50^

In summary, the contributions of this work are as follows.

• An end-to-end deep learning segmentation model for graph represented images using spline convolution layers is proposed.
• A comparison of the proposed GFCN and other methods in the literature for the prediction of acute stroke lesion in the ISLES2018 dataset challenge is given.

In Secs. 2.2 and 2.1, we discuss the dataset, preprocessing, evaluations approaches, and model architecture. In Sec. 3, we present the results of the ablation study to understand the function of different components of our algorithm. In addition, we unfold the results of the comparison of our model against the mentioned models. Finally, in Secs. 4 and 5, we discuss and present our conclusions.

## 2 Materials and Methods

In this section, we describe the network architecture in terms of the different depth configurations and the pooling and unpooling methods used. Next, we present the dataset, preprocessing, and evaluations approaches that were developed.

### 2.1 Network Architecture

The model used in this work has a similar architecture to the FCN in Ref. 46. We consider three variants, FCN-32s, FCN-16s, and FCN-8s, which differ in the way that skip connections are added to the upsampling path. The FCN-32s requires a 32× upsampling after five pooling layers of half-steps. The FCN-16s uses skip connections fusing features from previous layers by element-wise addition and requires a 16× upsampling because the output comes from four pooling layers of half-steps. Similarly, the FCN-8s uses a two-skip connection, so the output requires a 8× upsampling step, here the final output comes after three pooling layers of half-steps. In general, the FCN is built from local operations: convolution, pooling, and deconvolution. The deconvolution reconstructs a fine pixel a representation out of a coarser pixel structure. Subsequently, the network is divided into two parts: the downsampling path and the upsampling path.

• The downsampling path extends the receptive fields, increasing the contextual information in the next convolution layer; this is accomplished by the pooling layer. In the Euclidean case, the pooling uniformly reduces the pixel indices, increasing the receptive fields for the next convolutional layers. Conversely, in the case of the graph model, the pooling reduces the number of nodes and alters the topology of the network to a coarser and non-uniform grid. In our case, the coarsening is applied after every two concatenated convolutional layers. Each convolutional block doubles the feature dimensionality.
• The upsampling path redistributes the features to their previous location and recovers the initial node topology. The number of skip connections defines three variants of the FCN. The skip connections transfer local information to the forward layers by summing the previously generated features to the upsampled outputs; this helps to bring contextual information and to define the location of objects.

**Fig. 1:**
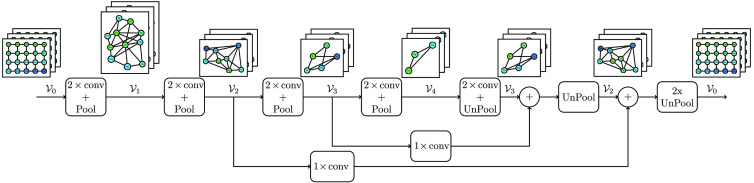
Direct graph of the GFCN-8s architercture. The notation $i,V_{j}$ represents a graph topology level j with i feature channels.

The FCN architectures are implemented using equivalent graph-based components, namely spline convolution filters,^32^ graph-based pooling operators, and two graph-based unpooling operators, described in Sec. 2.1. Figure 1 shows the FCN-8s variant of the FCN with graph operations. These variants are denoted GFCN.

• *GFCN-32s*. In this architecture, upsampling is built forward, reverting the coarsening operations without any skip connection.
• *GFCN-16s*. In this architecture, upsampling sums the output from the second pooling with graph topology $V_{2}$ to the upsampled transformation from the layer of the last downsampling path. Next, a direct upsampling to the input topology $V_{0}$ is applied.
• *GFCN-8s*. This architecture upsamples the prediction in three steps. In the first step, the unpooling is performed on the output of the last layer of the downsampling path, i.e., the pooling topology $V_{4}$. Next, it processes the sum of the last result and pooling topology $V_{3}$. Finally, it makes the double unpooling of the sum of the last result and the pooling topology $V_{2}$ to the input topology $V_{0}$.

The argument to use upsampling operators has the same meaning as in the Euclidean domain, where upsampling allows for recovering the initial dimensionality of the input and creating the segmentation map. In the case of the GFCN, the downsampling path and upsampling path have a non-uniform field of view expansions and contractions, which might help with the problem of insufficient localization of long-range features in the standard Euclidean CNNs as described in Ref. 51. This is also implemented in Ref. 45, though we explore two different ways to recover the features: the first approach simply copies the values to the neighbor nodes and the second approach distributes the values proportionally to their feature value in previous pooling topologies. The latter looks for having a perfect reconstruction of the previous feature space. However, proportional unpooling will be an approximation because we employ a max function as aggregation in the pooling layer, and a perfect reconstruction will only have an effect if average pooling was used. We decide on maxpooling because average pooling introduced vanishing gradients during training in our preliminary experiments; we do not report any results of those experiments in this manuscript.

#### 2.1.1 Pooling operators

The pooling layer reduces the number of nodes aggregating sets of similar nodes and applying a symmetry invariant operator, in this case, the max operator. Therefore, the pooling operation in layer l is done in two steps: first, a clustering forms a subset $V_{c_{i}}\subseteq V_{l}$ and the second step aggregates them with the max operator $\max(V_{c_{i}})$ to form the feature of the node $v_{i}\in V_{l+1}$ in the next layer. We explore three pooling approaches (i) the Top-k pooling,^45^ (ii) the radius clustering of points selected by the farthest point sampling algorithm as in Ref. 50, and (iii) finally the Graclus algorithm.^52^

#### 2.1.2 Unpooling operators

The unpooling operators restore the previous graph topology. We propose two approaches: the isotropic operator and the “proportional unpooling operator.” As a benchmark, we also use the KNN unpooling operator from Ref. 50, which computes the weighted sum of K neighbor nodes in the current layer to the node in the next layer. The weights are inversely proportional to the square distance of the nodes.

**Fig. 2:**
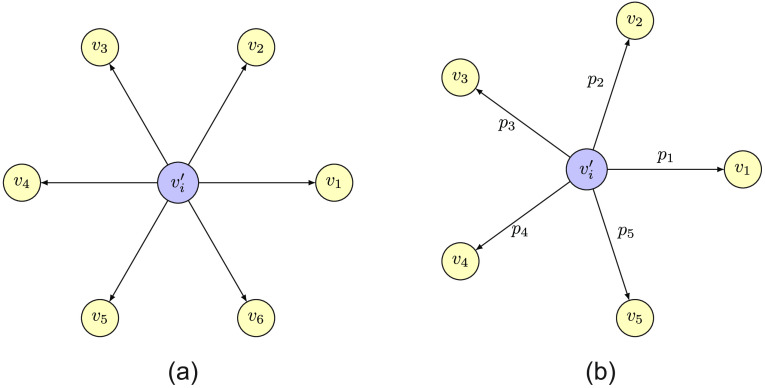
Representation of the unpooling approaches isotropic and proportional. (a) Isotropic approach. The features are copied to the position of the vertices that were aggregated into $v_{i}^{\prime }$, namely the set $V_{c_{i}}=\{v1,v2,v3,v4,v5,v6\}$. (b) Proportional unpooling operation. The features are weighted by a factor $p_{i}$ for i∈[1,5] to the position of the vertices that were aggregated into $v_{i}^{\prime }$, namely the set $V_{c_{i}}=\{v1,v2,v3,v4,v5\}$.

The “isotropic unpooling operator” copies the features in the positions of the previous nodes $v_{i}\in V_{c_{i}}$ that were aggregated into the target node $v_{i}^{\prime }\in V_{l}$; this is shown in Fig. 2, where the values from the target node topology $v_{i}^{\prime }\in V_{l}$ in layer l are copied to the position of the nodes $v_{i}\in V_{l+1}$ of the next layer. For that, we store the pooling assignment $V_{c_{i}}$ for node $v_{i}\in V_{l}$ in the target topology, where $V_{c_{i}}\subseteq V_{l-1}\equiv V_{l+1}$; hence we write

$$v_{i}^{\prime }=v_{i}\;\forall \;v_{i}^{\prime }\in V_{c_{i}}.$$ (1)

The proportional unpooling operator applies a factor $p_{i}$ that weights the feature propagation proportional to the sum of all members of the cluster $V_{c_{i}}$; then the propagation is written as

$$v_{i}^{\prime }=p_{i}\;v_{i}\;\forall \;v_{i}^{\prime }\in V_{c_{i}}\;\text{with}\;p_{i}=\frac{v_{i}}{\underset{v_{j}\in V_{c_{i}}}{\sum }v_{j}}.$$ (2)

The operations are computed without calculating the gradient of the weights $p_{i}$ to reduce the GPU memory, and the gradient is preserved in the upsampled node $v_{i}^{\prime }$, i.e., $p_{i}$ is independent of the weights.

### 2.2 Data and Preprocessing

#### 2.2.1 ISLES2018 dataset

We use the dataset from the challenge for stroke lesion segmentation, ISLES2018, which consists of CTP images within 8 h after the stroke episode, and a DWI within 3 h after the CTP was performed. The dataset consists of the perfusion parameter maps: cerebral blood flow, cerebral blood volume, mean transit time, and time to peak (Tmax). The original partition has 94 samples for training with mask information and 63 samples for testing without mask information. In the comparison experiments, we use a 3:1 rate training and testing cross-validation scheme out of the 94 cases with a mask. The training set in the cross validation is additionally split into a 9:1 rate for unbiased best model selection. Thereby, the final dataset splits use 65 cases for training, 6 for validation, and 23 for testing.

#### 2.2.2 Preprocessing

Preprocessing is considered a critical step in training the model. We use as inputs the structural CT and the CTP parameters, as well as a min–max instance normalization in each volume in a similar line as in Ref. 53. In the case of the CT, we enhance the contrast of the brain using a mask on the non-zero values of the sum of the CTP-parameters for each sample, in a similar way as in Ref. 28. No augmentation method was employed.

#### 2.2.3 Evaluation

We train the networks in the ablation study for 100 epochs equivalent to 4500 optimization steps with a batch of 4. We train most cases with different learning rates to ensure convergence, with the exception of variations of model architectures in which the learning rate was kept constant, as we want to evaluate the convergence speed due to the increment of features or the placement of the batch normalization layer. The training is done in a GEFORCE RTX 2080 TI with 11 GB of memory, an Intel^®^ Xeon^®^ CPU E5-2665 0 at 2.40 GHz, and 124 GB of RAM. The qualitative analysis considers the calculation of the Dice coefficient score (DCS):

$$\mathrm{DCS}=\frac{2\mathrm{TP}}{2\mathrm{TP}+\mathrm{TN}+\mathrm{FN}},$$ (3)

where TP, TN, and FN stand for the cardinality of the sets of true positive, true negative, and false negative voxel sets corresponding to a given segmentation $\hat{Y}$ with respect to a ground truth Y. In addition, the Hausdorff distance (HD), recall, precision, and coefficient of determination (COD) are computed:

HD(Y,Y^)=max{supy∈Y‖y,Y^‖,supy^∈Y^‖y^,Y‖}, (4)

$$\text{recall}=\frac{\mathrm{TP}}{\mathrm{TP}+\mathrm{FN}},$$ (5)

$$\text{precision}=\frac{\mathrm{TP}}{\mathrm{TP}+\mathrm{FP}},$$ (6)

COD=1−∑(y^−y)2∑(y^−E[y])2. (7)

The models are trained under a fourfold cross-validation regime with splits for training, validation, and testing of 65, 6, and 23, respectively. The best model trained in the training set is selected using the validation set, and the metrics reported correspond to the unseen samples on the testing set. The significance of comparisons is done with a pair t-student test on the testing set.

We compute the metrics DCS, accuracy, recall, precision, HD, and COD on average for every slice in the validation set after each epoch. The calculation is done in a sample-wise manner, meaning that the values are averaged over all slices in one batch and then averaged on the whole dataset. These values were relatively small because using 2D slices makes some of them have a mask of zero, which makes the values drop. We cope with this by calculating the evaluation metrics at the end of the training in the testing set in a case-wise (volume-wise) manner, i.e., we consider all of the voxels of a corresponding case volume in the dataset and then average these values for all cases in the dataset.

## 3 Results

We structured our study in two parts. First, we made a component evaluation in which we investigated different elements of our model and the correlation with the segmentation results. The second part of the study compared the performance of our model against other representative deep learning methods in the literature.

### 3.1 Ablation Study: Understanding Model Components

We have three degrees of freedom in the design of the model: (i) the architecture variants with different depths, i.e., 32s, 16s, and 8s; (ii) the unpooling operators, proportional and isotropic; and (iii) the pooling operators, Top-k, and Graclus. Therefore, the first experiments aim to find which is the best architecture and which is the best pooling and unpooling operators combination for the proposed GFCN model. Accordingly, the evaluation procedures are divided into two parts. The first part deals with the architecture configuration question and the second with the pooling and unpooling question, as described below.

#### 3.1.1 Model architectures: down-sampling depth, batch normalization, and skip connections

The first experiment compares the model architectures GFCN-8s, GFCN-16s, and GFCN-32s by training on the ISLES2018 challenge dataset (cf., 2.2) over 100 epochs with a constant learning rate of $1\times 10^{-6}$ with soft-Dice-loss as the optimization criterion.^54^ The training performance of the three models is compared using the same pooling and unpooling methods for all trials. We used Graclus pooling and isotropic unpooling. In addition, we investigate the placement of a batch normalization layer before and after the activation layer.^55^

The results in Table 1 show the performance metrics on the testing set, in which the best performing architecture is the GFCN-8s that uses batch normalization before the activation functions. The GFCN-16s shows lower performance than the GFCN-8s, which suggests that a deeper initial convolutional layer is necessary to extract better descriptors. In addition, comparing the GFCN-32s and the GFCN-16s cues the positive effect of skip connections. The GFCN-16s uses a skip connection from the pool-score of the $V_{3}$ graph topology, whereas the GFCN-32s is devoid of forwarding loops. We observe this effect as much for pre-batch normalization (pre-BN) as for post-batch normalization (post-BN).

**Table 1:** Comparison of model architectures on the ISLES2018 challenge dataset for segmentation of ischemic stoke lesions. Metric calculated per volume in average for 23 testing samples.

Arch.	DCS	Accuracy	Precision	Recall	HD	COD
Pre-BN						
GFCN-32s	0.2543 ±0.0001	0.9186 ±0.0004	0.1652 ±0.0001	0.8839 ±0.0013	100.5624 ±62.9937	−31.7704 ±218.3604
GFCN-16s	0.2827 ±0.0001	0.9350 ±0.0001	0.1911 ±0.0001	0.8388 ±0.0014	98.8227 ±79.5752	−22.2204 ±149.2124
GFCN-8s	**0.3962** ±**0.0012**	**0.9749** ±**0.0001**	**0.3583** ±**0.0035**	0.6363 ±0.0190	**73.2866** ±**123.5770**	−**3.6425** ±**0.7375**
Post-BN						
GFCN-32s	0.1979 ±0.0003	0.8762 ±0.0009	0.1222 ±0.0002	**0.9350** ±**0.0009**	104.9627 ±42.9018	−38.6241 ±303.6866
GFCN-16s	0.2700 ±0.0001	0.9300 ±0.0002	0.1818 ±0.0001	0.8308 ±0.0035	99.8393 ±60.0495	−22.1760 ±115.7197
GFCN-8s	0.3654 ±0.0005	0.9697 ±0.0001	0.3069 ±0.0012	0.6473 ±0.0055	80.2424 ±69.8212	−5.8978 ±7.6697

Best performance in bold.

**Fig. 3:**
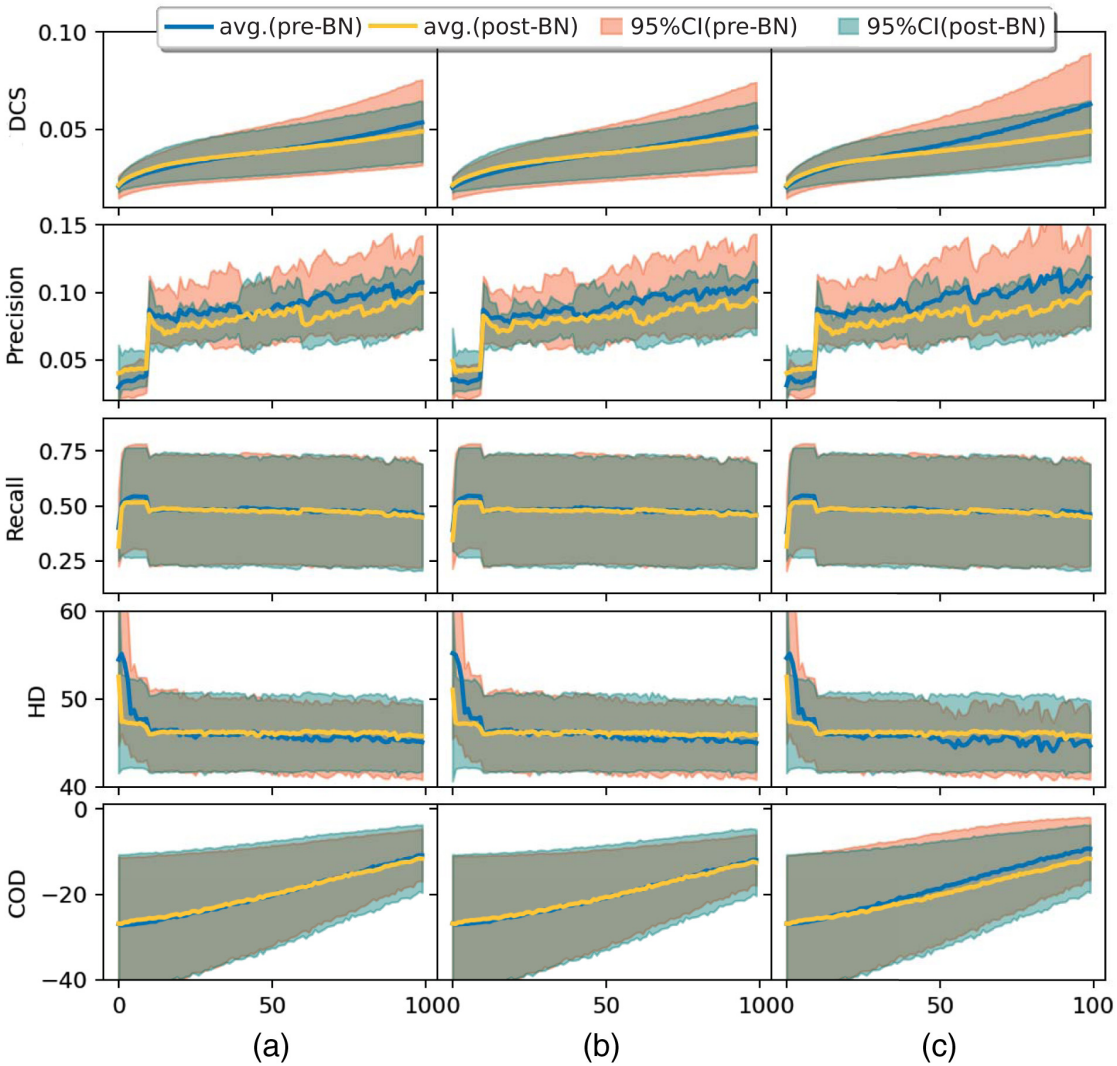
Validation metrics (DCS, precision, recall, HD, and COD) per epoch for the ISLES2018 challenge dataset comparing the GFCN architectures (a) GFCN-32s, (b) GFCN-16s, and (c) GFCN-8s ordered by columns. Metrics calculated in a sample-wise manner in the validation set (six samples, being a sample 3D volumes with many 2D slices). Lines: blue avg. (pre-BN) correspond to average metrics using pre-batch normalization; and orange avg. (post-BN) correspond to average metrics using post-batch normalization. The areas: pink 95%CI (pre-BN) correspond to 95% confidence intervals for metrics using pre-batch normalization; and green 95%CI (post-BN) correspond to 95% confidence intervals for metrics using post-batch normalization.

Figure 3 shows that the three architectures continue improving after 100 epochs; still, the GFCN-8s with deeper initial feature extraction improves more quickly than the other two architectures. Regardless of the post-BN starting at a higher value than the pre-BN, the velocity of convergence increases in the pre-BN case. This is consistent across the architecture variations, though the difference increases with a deeper architecture. For example, this can be observed by comparing the differences between pre-BN and post-BN in the GFCN-8s or GFCN-32s.

#### 3.1.2 Pooling and unpooling methods

The second experiment compares the pooling and unpooling methods. The model architecture used is the GFCN-8s trained from scratch on the ISLES2018 dataset for 100 epochs with early stopping. Again, the learning rate is constant. We collect the metrics after each epoch sample-wise, and at the end of the training, we evaluate volume-wise on the 23 testing samples. We defined four variants of the models that combine compatible operators, i.e., for pooling: Graclus and Top-k;^45^ and for unpooling: isotropic, proportional, and k-NN interpolation of Ref. 50. The isotropic and proportional unpooling employ the Graclus pooling layers, as detailed in Sec. 2.1; and in the case of the Top-k, it uses the k-NN interpolation as the pooling method. Finally, we included one model that uses no pooling operators. This adds up to four models studied in this experiment: isotropic, proportional, Top-k, and no-pooling.

**Fig. 4:**
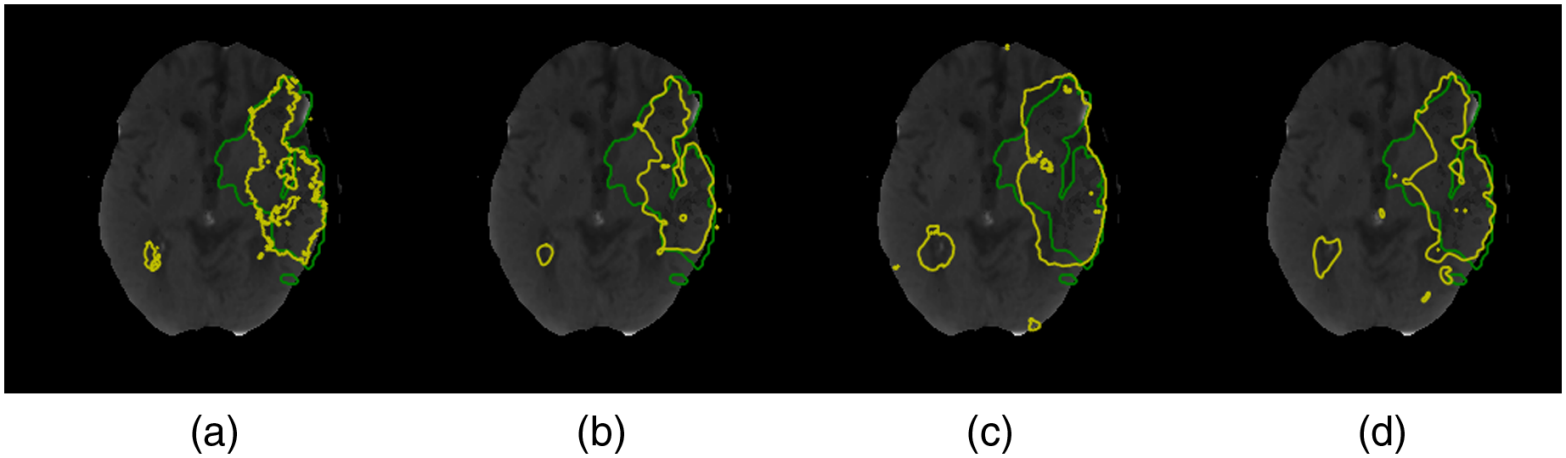
Segmentation contour results for the ISLES2018 challenge dataset comparing (a) upsampling operations isotropic, (b) proportional, (c) Top-k, and (d) no-pooling. The ground truth is in green, and the segmentation probability with a threshold of 0.5 is in yellow.

The results presented in Table 2 show the performance metrics of four variants of pooling and unpooling layers for a fixed architecture GFCN-8s. We observe that performance of the isotropic and proportional upsampling remains in a similar range. In all trials, the HD is lower for the isotropic pooling than for the proportional pooling (2.04%<5.0% p-value). This is consistent with what is shown in Fig. 4, where the boundaries obtained with the isotropic are closer to the ground truth, though segmentation probabilities are smoother in the proportional upsampling. It is worth noting that the isotropic approach is less computationally expensive than the other approaches, which translates into less training time. Top-k and no pooling generally have better metrics than the proposed upsampling methods but considerably less sensitivity (0.43%<1.0% p-value).

**Table 2:** Upsampling methods comparison on the ISLES2018 challenge dataset. Isotropic stands for the isotropic unpooling operator. Proportional stands for the proportional unpooling operator. Comparisons of fixed GFCN-8s architecture with batch normalization before activation, trained over 100 epochs.

Unpooling	DCS	Accuracy	Precision	Recall	HD	COD
Isotopic	0.3962 ±0.0012	0.9749 ±0.0001	0.3583 ±0.0035	0.6363 ±0.0190	73.2866 ±123.5770	−3.6425 ±0.7375
Proportional	**0.4137** ±**0.0041**	0.9713 ±0.0001	0.3276 ±0.0068	**0.7907** ±**0.0060**	101.0747 ±90.2888	−17.2369 ±75.5638
Top-k	0.3432 ±0.0034	0.9833 ±0.0000	**0.4563** ±**0.0066**	0.3855 ±0.0255	**60.1466** ±**228.9974**	−0.8958 ±0.3723
No-pooling	0.4123 ±0.0022	**0.9808** ±**0.0000**	0.4157 ±0.0039	0.5512 ±0.0144	76.0657 ±124.6328	−1.7695 ±1.3348

Best performances in bold.

### 3.2 Performance Comparison with Other Methods

In the second part of the experiments, we contrast the proposed model with existing models for semantic segmentation. Consequently, we train various models in the literature from scratch using the same inputs as we use for the proposed model, namely the FCN-8s,^46^ U-Net,^49^ and PointNet++.^50^

The evaluation is done with fourfold cross validation in the ISLES2018 dataset with the same splits as the one for the GFCN-8s. In all experiments, the models are trained for a maximum of 300 epochs with early stopping to avoid overfitting. The learning rate is reduced after 100 epochs by a factor of 10. We train the models using an Adam optimizer and a soft Dice loss.^54^ The proposed model, denominated GFCN-8s, uses Graclus pooling and isotropic upsampling layers.

The results in Table 3 showed better metrics for the GFCN-8s compared with the other models trained under the same configuration. FCN-8s has the second best values among these four models. It is worth noticing that the used FCN-8s model is not exactly the model from Ref. 46 but a simplification with a similar architecture to the GFCN-8s. This was adopted because the low number of training samples would be insufficient to train all weights in the original configuration. In the case of the U-Net, we use a bilinear interpolation layer for the upsampling instead of a learnable deconvolution layer, but we include batch normalization. Despite these differences, we preserve the original U-Net architecture in Ref. 49. Finally, in the case of the Pointnet++, we employ the same configuration described in the original work.^50^

**Table 3:** Comparison of segmentation models on the ISLES2018 challenge dataset. 2D-ARED stands for 2D asymmetric residual encoder–decoder from Ref. 28. COD and accuracy are not reported in Refs. 29, 28, and 27 in the original papers, as well as the dataset partitions. We simply exclude these values in the comparison.

Approach	DCS	Accuracy	Precision	Recall	HD	COD
U-Net	0.3821 ±0.0000	0.9456 ±0.0070	0.3631 ±0.0146	0.3833 ±0.0142	87.3667 ±1270.2201	−13.6742 ±36.8269
FCN-8s	0.4177 ±0.0007	0.9816 ±0.0000	0.4731 ±0.0159	0.4079 ±0.0025	54.8429 ±28.9919	−1.9943 ±4.9677
PointNet++	0.2216 ±0.0007	0.9630 ±0.0016	0.3011 ±0.0276	0.2728 ±0.0056	89.8277 ±646.9835	−2.9903 ±23.6567
GFCN-8s (ours)	**0.4553** ±**0.0031**	**0.9864** ±**0.000**	**0.4916** ±**0.0007**	**0.4447** ±**0.0130**	**62.3893** ±**13.0795**	−**1.5305** ±**0.8267**
3D U-Net^29^	0.5144	—	0.4737	0.7065	34.7591	—
2D-ARED^28^	0.5470 ±0.242	—	0.578 ±0.291	0.609 ±0.25	23.5 ±15.8	0.82
SLNet^27^	0.6211 ±0.1718	—	0.6197 ±0.2198	0.6952 ±0.1789	19.27 ±15.05	—

In addition, we append the results reported in Ref. 27 on the SLNet, a 2D-patch-based U-Net presented in Ref. 28, and the 2018 winner algorithm with a 3D U-Net reported in Ref. 29 as reference. Comparing the GFCN-8s against the external models, we notice that a considerable difference exists in the metrics unfavorable to our model, which is especially important in DCS and HD. Notice that the models reported in these external references are extensively optimized and employ complex feature extraction pipelines, special arrangements of convolutional layers and/or advanced augmentation methods. We stay in a simple input configuration and employ no augmentation methods because we are solely interested in understanding the process of graph CNNs for detecting stroke lesions.

**Fig. 5:**
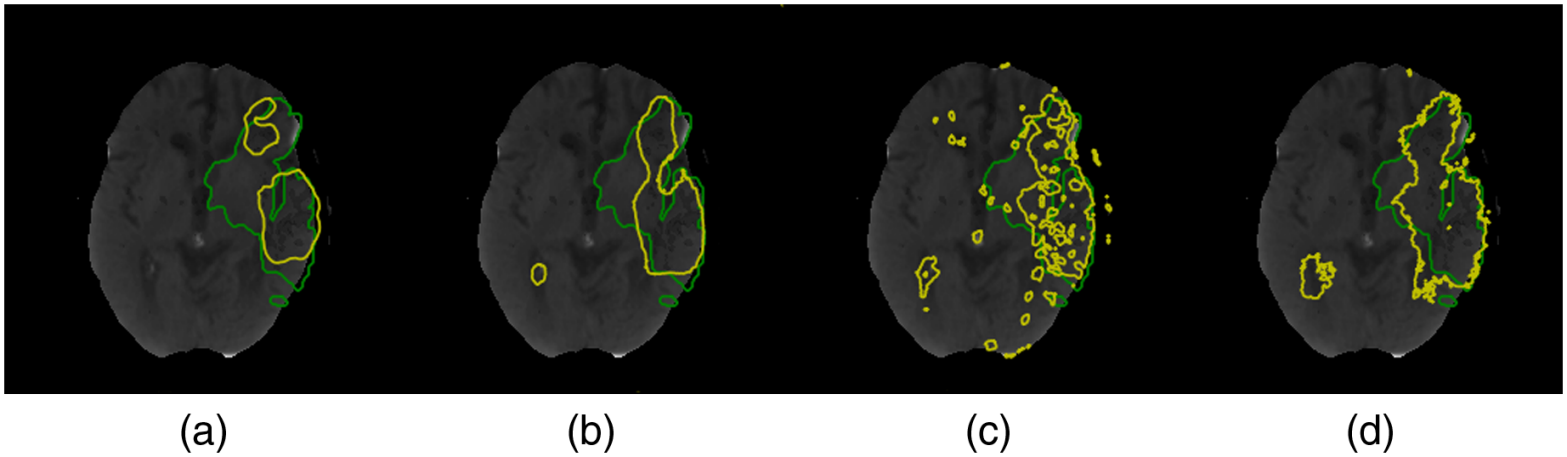
Comparison of segmentation mask generated with 0.5 probability threshold for models trained in the ISLES2018 challenge dataset, namely (a) U-Net, (b) FCN-8s, (c) PointNet++, and (d) GFCN-8s. The contour lines of the mask are in yellow, and the ground truth segmentation mask is in green.

Figure 5 shows a comparison of the segmentation boundaries for the trained models. The PointNet++, despite being able to successfully capture small structures, has several regions of false positives and therefore has the lowest average accuracy. Comparing the FCN-8s and U-Net, we notice that a deeper model will require more samples to train and refine the prediction as the U-Net successfully localizes the lesion but fails to correctly define the boundaries. The U-Net tends to produce fewer false positives, but it is less sensitive. On the other hand, the results of the FCN-8s are similar to the proportional unpooling from the previous experiment. The FCN-8s extracts smooth probability maps, as depicted in Fig. 5, but the prediction misses reaching the edges of the lesion. In contrast, GFCN-8s has a very flexible prediction output, and regardless of having a lower precision, compared with additional external models, it has the best average metric values among the models that we trained.

**Fig. 6:**
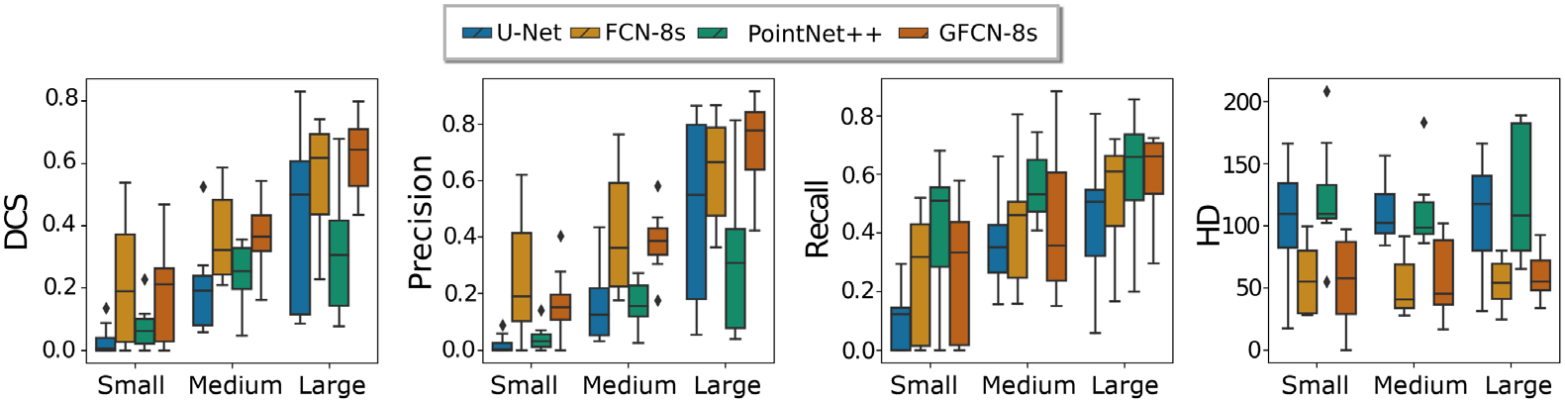
Comparison of the distribution of metric values along lesion size volume percentage.

Figure 6 shows the distribution of metric values calculated volume-wise and stratified into three categories according to the lesion’s volume, namely small, medium, and large lesion sizes. The sets are constructed from the distribution of the number of lesion voxels per scan split into three evenly distributed groups using a quantifier discrete cut. We observe that the PointNet++ is able to collect a higher number of lesion masks consistently along with the distribution of sizes, which leads to high recall values, yet it has the lowest precision values. The proposed GFCN-8s scores higher values compared with the other models trained. A trend of the lesion size in which smaller lesions have worse values than bigger lesions is also noticeable. This is consistent in all of the trained models.

## 4 Discussion

Our study focuses on identifying the relevance of GFCN in solving the segmentation problem of acute ischemic stroke lesion prediction and investigating the behavior of its components concerning the segmentation results. In this section, we analyze the results concerning the evaluation of different model architectures based on the number of convolutional blocks, pooling/unpooling operations, and batch normalization. In addition, this section also covers the comparison with selected methods trained under the same regime and state-of-the-art methods.^27^^–^^29^ This section discusses, in particular, the significance and limitations of the experiments, as well as points out recommendations for future research.

### 4.1 Understanding Model Components

#### 4.1.1 Feature extraction and perception filters

Including more convolutional blocks in the downsampling path improves the feature extraction and as a consequence the segmentation results. In the early days of deep learning, Jia et al.^56^ showed that spatial pooling allows for constructing overall semantics from the low-level features in analogy to the biological mechanics of the mammalian visual cortex.^57^ Moreover, images subject to inner and outer scale information exhibit the property of being invariant to small spatial shifts.^58^ Research suggests that in CNNs this property comes from the denominated upscaled receptive fields.^59^ The coarsening of the output of a convolutional layer expands the receptive field by a factor equal to the stride, as explained in the FCN original work.^46^ Further, the pooling factors allow for effectively calculating gradients when the receptive fields overlap. Therefore, the model with a deeper downsampling path has faster improvements and better metric values on their prediction values due to their increased receptive fields.

From the analysis, it is difficult to unequivocally identify the feature propagation across nodes. However, the irregular and close adaptation to the edges of lesions might suggest a flexible feature projection, yet we do not provide enough evidence to support this. Further research should be conducted exploring the perception fields and activation maps as in Refs. 60 and 61.

#### 4.1.2 Pooling and unpooling methods

Different pooling and unpooling approaches lead to vast differences in the segmentation results. This is shown in the differences in metric values as well as segmentation boundaries with and without pooling operations. Albeit the results obtained with the model “without pooling (no-pooling)” have smooth boundaries and by itself the spline CNN allows for extracting local information, the perception field does not change, which led to diminished performance. In Ref. 34, their model also used a spline CNN convolution layer and no pooling, so they also showed that it is possible to obtain good results. However, we found that the model was more difficult to train due to the high computational requirements during the calculation of gradients. Therefore, we show that pooling plays a major role and it is important to efficiently compute the predictions.

Simple heuristic upsampling approaches, such as the isotropic or proportional upsampling, which are independent of the gradient of the model, obtain comparable results to optimized approaches with fewer complications during training. For example, considering the case of the denominated Top-k model, we found that it is rather unstable and prone to vanishing gradients. This problem might be due to the dependence of gradients and the inline optimization of neighbors. In fact, the learnable projection of the Top-k pooling voids the pixel location conversely to the classical 2D pooling scheme; though, to some degree, this might be compensated by the spline-CNN, the optimization remains difficult. In the original work,^45^ this is not an issue because the local information is not relevant for their problem. As a result, we might expect that heuristic upsampling can reach a more stable and efficient optimization in other segmentation problems.

#### 4.1.3 Batch normalization

As expected, the placement of batch normalization before the activation function evinced a faster optimization curve than placing it after the activation function; this is shown in Fig. 3 and Table 1, where the batch normalization placement effect was compared. Thereby, the position of normalization in graph networks is consistent with what is stated in Ref. 55 as anticipated. This might imply that, by placing the batch normalization before the activation, different neurons will activate due to a change of sign induced by the non-linearity during the training. By contrast, by placing the batch normalization after the activation, the first and second momenta will not affect the sign. Therefore, it can be inferred that the output of the spline-convolutions have a symmetric non-sparse distribution as with the Euclidean case, as stated in Ref. 55.

As a limitation, it is worth mentioning that, by comparing the different architecture configurations, the results shown in Fig. 3 are not at the end of the optimization. We aimed to compare the convergence with a simplified hyperparameter configuration; therefore, we fixed the learning rate and optimization steps. However, this does not void the results shown in Table 1 because the optimization curvature will normally tend to reduce and we should not expect major changes in the optimization trends. In addition, due to the high dimension of the feature spaces, the topology of the optimization will not differ substantially as distances are small.^62^ Further, the models start at the exact same optimization points as shown in Fig. 3.

### 4.2 Comparing GFCN with Other Methods

Smaller lesions present a more difficult challenge than bigger lesions. The analysis of the distribution of metrics by size revealed that samples with medium and large sizes have better metric values than smaller samples, which is consistent with what is reported in the literature.^19^ This might be explained by the fact that small size lesions are associated with class imbalance. Small lesion size has somehow blended with the background inherited by the CT acquisition and resolution limitations, reported in Refs. 53 and 63. Smaller lesions will tend to blend with the background signal surrounding the lesion itself.

The GFCN obtained lower metrics compared with state-of-the-art models,^27^^–^^29^ with absolute differences for the DCS metric ranging from 12.18% to 30.80%. It is probable that the proposed model would reach comparable metric values in a more optimized input setup. On the other hand, the GFCN obtained better metrics than the models trained under the same conditions, with absolute differences for the DCS metric ranging from 8.61% to 69.05%. The difference between the results of U-Net and FCN-8s is in line with research showing that big models require large amounts of data.^25^^,^^64^ Therefore, the surprisingly low metric values for the U-Net compared with the FCN-8s might be explained by the simple input and training setup adopted, as the training of bigger models require augmentation.^20^^,^^65^ Despite this fact, the comparison is still valid as we wanted to keep the same simplified training environment. As an outlook, a way to cope with the low number of samples could be to use a patch-wise training approach as in Ref. 28 or the generative model approach as in Ref. 27.

### 4.3 Medical Implications

The similar behavior of our model in the results of lesion size stratification suggests that our model would obtain similar salient and activation maps as a standard U-Net, and thus we could extrapolate the results of Ref. 31; even so, further study is required to understand these maps with the graph layers of our model. In medical practice, the volume of the lesion can be calculated from the segmentation output of our model, although it is possible that the model will overlook small lesions. An explanation for this can be found in Ref. 31, where bigger lesions generate a larger response in the neural network than small lesions, which implies that these small volumes get lost in signals through the layers of the network.

The studies^66^^–^^68^ prove the diagnostic power of penumbra regions extracted from CT perfusion and CTP-parameters in correlation with scored studies, such as Alberta Stroke Program Early CT Score, National Institutes of Health Stroke Scale, or modified Rankin Scale. The same outputs can be rapidly predicted for new unseen CTP inputs with up to 0.4553 DCS because the most time-consuming and complex process of training the model is completed at this time point. Patients will require a non-contrast CT and CTP, with the calculation of the CTP-parameters. The volumes require a simple min–max normalization calculated directly for each volume at a low computational cost.

In addition, the proposed model is useful in the assessment of penumbra in cases in which the onset time is undefined in line with the findings in Refs. 69 and 67. It is shown that the determination of penumbra allows for assessing the neurological deficit and infarctic volume in patients for which the time of stroke intake is unknown. Other studies have shown that, by measuring the size of the penumbra and computing the core/penumbra rate, it is possible to identify candidate patients for rtPA perfusion within the 6 h window upon stroke intake as it is demonstrated that these patients might have an improved outcome compared with placebo patients. Therefore, the proposed model in combination with the regression models proposed in Ref. 67 could potentially allow for predicting, for example, the NIHSS at 7 days after admission.

## 5 Conclusions

In this study, we focused on understanding the principles governing a graph-based FCN to estimate irreversible brain stroke lesions and to see how they differ from classical Euclidean models. Based on the ablation experiments, we observed changes in the results with deeper networks, in which more convolutional blocks enhanced the segmentation results. Furthermore, an overall view of the segmentation results showed that feature propagation in the reception fields developed into an irregular and closer adaptation to the edges of the lesion, evincing the effect of inter-pixel features. With regard to the different pooling and unpooling approaches used, we noticed that they lead to visible differences in the segmentation results (cf., Fig. 4), where simple heuristic approaches allow for fewer features and therefore less computation. In general, the model could be used in medical practice, but it will overlook small lesions. In comparison with other methods, we found that smaller lesions were more difficult to identify than bigger lesions, which is consistent with the literature. The evaluation of our model against models trained in the same regime showed that our model performed better for the metrics reported; for example, in the case of the DCS metric, we obtained improvements ranging from 8.61% to 69.05%. However, the training approach can be improved by changing the inputs to precomputed DWI from generative models as in Ref. 27 or using patch-wise training as in Ref. 28. Moreover, it might be advantageous to restructure the proposed architecture, for example, to introduce a learnable deconvolution as in Refs. 70 and 71. In addition, further visualization methods, such as activation maps or salient maps as in Refs. 39 and 61, could help to understand better the internal process of the model. The activation maps are particularly important for validating and assessing prediction in medical applications.^72^^–^^74^ In addition, they could shed some light on characteristics of the feature propagation across the field of view within the layers of the GFCN.^75^^–^^77^

## Code, Data, and Materials Availability

The code is available at https://github.com/aiporre/gcn_segmentation
